# Doublecortin-Like Kinase 1 (DCLK1) Is a Novel NOTCH Pathway Signaling Regulator in Head and Neck Squamous Cell Carcinoma

**DOI:** 10.3389/fonc.2021.677051

**Published:** 2021-07-16

**Authors:** Esther C. Broner, Jonathan A. Trujillo, Michael Korzinkin, Tejaswini Subbannayya, Nishant Agrawal, Ivan V. Ozerov, Alex Zhavoronkov, Lisa Rooper, Nikita Kotlov, Le Shen, Alexander T. Pearson, Ari J. Rosenberg, Peter A. Savage, Vasudha Mishra, Aditi Chatterjee, David Sidransky, Evgeny Izumchenko

**Affiliations:** ^1^ Department of Otolaryngology and Head & Neck Surgery, Johns Hopkins University, School of Medicine, Baltimore, MD, United States; ^2^ Department of Medicine, Section of Hematology and Oncology, University of Chicago, Chicago, IL, United States; ^3^ InSilico Medicine Hong Kong Ltd., Pak Shek Kok, Hong Kong; ^4^ Institute of Bioinformatics, International Technology Park, Bangalore, India; ^5^ Section of Otolaryngology-Head and Neck Surgery, University of Chicago, Chicago, IL, United States; ^6^ Department of Pathology, Johns Hopkins University, School of Medicine, Baltimore, MD, United States; ^7^ Faculty of Bioengineering and Bioinformatics, Lomonosov Moscow State University, Moscow, Russia; ^8^ Department of Pathology, The University of Chicago Medicine, Chicago, IL, United States; ^9^ Manipal Academy of Higher Education, Manipal, India

**Keywords:** HNSCC, DCLK1, NOTCH, tumor microarray, transcriptomic analysis

## Abstract

Despite recent advancements, the 5 year survival of head and neck squamous cell carcinoma (HNSCC) hovers at 60%. DCLK1 has been shown to regulate epithelial-to-mesenchymal transition as well as serving as a cancer stem cell marker in colon, pancreatic and renal cancer. Although it was reported that DCLK1 is associated with poor prognosis in oropharyngeal cancers, very little is known about the molecular characterization of DCLK1 in HNSCC. In this study, we performed a comprehensive transcriptome-based computational analysis on hundreds of HNSCC patients from TCGA and GEO databases, and found that DCLK1 expression positively correlates with NOTCH signaling pathway activation. Since NOTCH signaling has a recognized role in HNSCC tumorigenesis, we next performed a series of *in vitro* experiments in a collection of HNSCC cell lines to investigate the role of DCLK1 in NOTCH pathway regulation. Our analyses revealed that DCLK1 inhibition, using either a pharmacological inhibitor or siRNA, resulted in substantially decreased proliferation, invasion, migration, and colony formation. Furthermore, these effects paralleled downregulation of active NOTCH1, and its downstream effectors, HEY1, HES1 and HES5, whereas overexpression of DCLK1 in normal keratinocytes, lead to an upregulation of NOTCH signaling associated with increased proliferation. Analysis of 233 primary and 40 recurrent HNSCC cancer biopsies revealed that high DCLK1 expression was associated with poor prognosis and showed a trend towards higher active NOTCH1 expression in tumors with elevated DCLK1. Our results demonstrate the novel role of DCLK1 as a regulator of NOTCH signaling network and suggest its potential as a therapeutic target in HNSCC.

## Introduction

Head and neck squamous cell carcinoma (HNSCC) is the seventh most common cancer worldwide, with approximately 890,000 new cases and 450,000 deaths associated with the disease (1). Etiologic risk factors for HNSCC include tobacco use, alcohol consumption, and infection with carcinogenic strains of human papilloma viruses (HPV) (2–4). Despite recent advances in diagnosis and treatment approaches, the survival rates for HNSCC have remained largely unchanged, with a 50% 5-year survival (5).

Although large-scale genomic and gene expression studies have provided insight into the mutational patterns and molecular pathways in the development of these tumors (6–8), few targets have translated into effective molecularly targeted therapies in the clinic. The identification of actionable drug targets for HNSCC has been hindered by the low frequency of targetable oncogene aberrations and the predominance of loss-of-function mutations in tumor suppressor genes, which are difficult to drug and correct directly (8). Thus, there is an urgent need to identify molecular drivers of HNSCC carcinogenesis that can be targeted therapeutically.

Doublecortin-like kinase 1 (DCLK1), a member of the protein kinase superfamily and the doublecortin family, regulates microtubule polymerization and is involved in neurogenesis and neuronal migration (9). Recently, DCLK1 has been identified as a potential cancer stem cell marker, and has been implicated in playing a functional role in carcinogenesis, promoting cancer initiation, tumor invasion, and metastases in several solid malignancies, such as pancreatic adenocarcinoma, colorectal cancer, and renal clear cell carcinoma (10–13). Furthermore, overexpression of DCLK1 is clinically associated with tumor progression and poor prognosis in several human cancers, including HNSCC (14–16).

In HNSCC, high expression of DCLK1 is associated with the development of recurrent or metastatic disease and higher mortality (17, 18). However, little is known about the molecular mechanisms underlying the role of DCLK1 in HNSCC tumorigenesis. Thus, additional functional studies are needed to further elucidate the oncogenic impact of DCLK1 in promoting the malignant potential of cancer cells and its role in the adverse prognosis noted in HNSCC.

In this study, we demonstrate that DCLK1 down-regulation in HNSCC cells by siRNA-mediated knockdown or through pharmacologic inhibition *in vitro* resulted in reduced cancer cell proliferation, migration, and cell invasion. Conversely, overexpression of DCLK1 in normal oral keratinocytes, resulted in increased cell proliferation. Furthermore, through transcriptome-based computational analysis of primary HNSCC tumors and *in vitro* experiments we showed that DCLK1 upregulates NOTCH signaling, an important driver in HNSCC carcinogenesis (19–21). High-expression of DCLK1 in HNSCC tumors was associated with reduced overall survival in our independent cohort of HNSCC patients as noted in one previous study (16). Overall, these data demonstrated that DCLK1 is functionally involved in HNSCC carcinogenesis and identified a novel role for DCLK1 as a positive regulator of the NOTCH signaling network. Mounting evidence, including these data, indicate that DCLK1 is an important oncogenic driver in a subset of HNSCC patients and may represent a potential therapeutic target.

## Materials and Methods

### Cell Lines

Human HNSCC cell lines (JHU-011, JHU-022 and JHU-029) we established at Johns Hopkins University, and were maintained in the RPMI medium containing 10% Fetal Bovine Serum (FBS), penicillin (100 units/mL) and streptomycin (100 μg/mL). SCC25, FaDu, Cal27, SCC25, and SCC22b cell lines were obtained from ATCC and grown in the recommended medium. Tert-1 immortalized normal human oral mucosal keratinocytes cell line OKF6 was received from Dr. James Rhienwald, Harvard Medical School, and spontaneously immortalized NOK-SI cell line was received from Dr. Silvio Gutkind, National Institute of Dental and Craniofacial Research. Cells were maintained in Keratinocyte-SFM (Thermo Scientific, Cat# 17005042). The cells were periodically monitored for mycoplasma using the MycoDtect kit (Greiner Bio-One). All experiments were performed within 6 months of the mycoplasma screen. All cells were incubated at 37°C with 5% CO2.

### Cell Viability Assay

Relative cell viability was determined using an Alamar Blue assay as outlined by the manufacturer (AbDSerotec, Cat# BUF012B). New media containing 1/10 volume of Alamar Blue reagent was added to the wells and cells were incubated at 37°C for 1 hour. Fluorescence (545 nm excitation, 590 nm emission wavelengths) was measured using a SpectraMax-Plus384 fluorometer. Cell viability was calculated relative to the control cells incubated in parallel.

### Wound Healing Assay

Cells were cultured in triplicate in 6-well plates (1×10^6^ cells per well) containing culture inserts (Ibidi, Cat# 80209). On reaching 90% confluence, the inserts were removed and cells were cultured for 12 hr. Gap area in individual wells were determined using ImageJ. The gap area percentage was calculated as the gap area at 12 hr relative to the gap area at 0 hr.

### Cell Invasion Assay

Cells were cultured (2.5×10^4^ cells per well) in triplicate into the upper chamber of Matrigel-coated transwell chambers (8-µm pore size, Corning, Cat# 354480) in serum-free medium, while 10% FBS medium was added to the bottom chamber to stimulate invasion. After 24 h incubation, the cells in the upper chamber were carefully removed with cotton swab and the cells that had invaded through Matrigel were stained with 1DiffQuick (RAL Diagnostics, Cat# 10736131), photographed at 40X magnification and quantified across three random fields.

### Immunoblotting

Protein lysates were prepared in RIPA lysis buffer (Thermo Scientific, Cat# 89900), and protein concentrations were measured by the bicinchoninic acid method (Thermo Scientific, Cat# 23225). Proteins from each sample were resolved by electrophoresis under reducing conditions in 4-12% Bis-Tris NuPAGE gels (Thermo Scientific, Cat# NW04120BOX) according to manufacturer’s instructions and transferred to PVDF membrane (EMD Millipore, Cat# IPVH00010). Membranes were blocked in Immobilon Signal Enhancer (Millipore Sigma, Cat# WBSH0500) and incubated with primary antibodies overnight at 4°C followed by incubation with HRP-linked secondary antibodies (Cell Signaling Technology, Cat# 7074 and 7076). Protein bands were visualized by chemiluminescence using the ECL Western blotting Detection System (GE Healthcare, Cat# 89168-782) or SuperSignal West Femto Maximum Sensitivity Substrate (Thermo Scientific/Pierce Biotechnology, Cat# 34094). The following primary antibodies were used: NICD1 (Cell Signaling, Cat# 4147S, MW 110 kDa), HES1 (Abcam, Cat# ab71559, MW 30 kDa), HEY1 (Millipore Sigma, Cat# AB5714, MW 33 kDa), HES5 (Novus Biologicals, Cat# NBP241305, MW 17 kDa), DCLK1 (Cell Signaling Technology, Cat# 62257, MW 82 kDa), p-DCLK1 (Abbomax, Cat# 630-620, MW 82 kDa) and GAPDH (Santa Cruz, Cat# sc-47724, MW 37 kDa). Note that according to the manufacturer an anti-DCLK1 antibody used in this study specifically detects the 82 kDa isoform (transcript variant 1) in human derived tissue and cell lines (22–26).

### DCLK1 Inhibition

DCLK1 inhibitor, LRRK2-IN-1 (Millipore Sigma, Cat# 438193-5MG), was diluted in DMSO and cells were treated with 5μM, 10μM and 20μM concentrations for 72-96h. Final concentration of DMSO did not to exceed 0.01%.

### RNA Interference

Three different 27mer siRNA duplexes targeting DCLK1 and Trilencer-27 Universal Scrambled Negative Control siRNA Duplex were purchased from OriGene Technologies (Cat# SR306088). This product was designed to target multiple splice variants generated by two alternative promoter usage and alternative splicing, including transcript variant 1 (NM_004734) and transcript variant 2 (NM_001195415). To deplete target gene expression three independent siRNA duplexes were pooled together and cell lines were transfected using Lipofectamine 3000 (Thermo Scientific, Cat# L3000008) according to manufacturer’s protocol.

### DCLK1 Overexpression

Plasmid pCMV6-AC-GFP containing human DCLK1 variant 1 ORF (NM_004734) which is fused with a turboGFP at C-terminal (OriGene Technologies, Cat# RG217050) and control empty vector were transfected into OKF6 and NOK-SI cells with jetOPTIMUS DNA Transfection Reagent (Polyplus transfection, Cat# 76299-630).

### cDNA Synthesis and Reverse Transcription-PCR (RT-PCR)

All predesigned qPCR assays were ordered from Integrated DNA Technologies (IDT). Hs.PT.58.19740848 assay was used for DCLK1 analysis. This assay recognizes transcript variant 1 (NM_004734) and transcript variant 2 (NM_001195415). RNA extraction and cDNA conversion were performed by Trizol reagent (Thermo Scientific, Cat# 15596026) and Quantitect Reverse Transcription Kit (Qiagen, Cat# 205313) respectively following manufacturer instruction. Gene products were amplified using iTaq SYBR green Supermix with Rox dye (Bio-Rad Laboratories, Cat# 1725120). All reactions were performed in triplicate and relative quantity was calculated after normalizing to GAPDH expression.

### Formalin-Fixed Cell Pellet Blocks

JHU-011 cells were harvested by trypsinization, washed twice in PBS, resuspended in 10% neutral buffered formalin (NBF), and pipetted into a 0.6-ml microfuge tube containing 200 μl of solidified agarose (2% in PBS). The microfuge tube was centrifuged at 200 × *g* for 5 minutes at room temperature to form a uniform cell layer on the agarose bed. Supernatant was aspirated, and pellet was carefully overlaid with 200 μl 10% NBF. After repeating centrifugation, the microfuge tube was carefully submerged in 10 mL 10% NBF solution for 24–48 hr. The fixed pellet was then removed, processed, and paraffin embedded.

### Tissue Microarray and IHC

Tissue microarrays (TMAs) containing tumor cores from 233 newly diagnosed and 40 recurrent patients with HNSCC were obtained under Institutional review board–approved protocols from Johns Hopkins University. TMA slides as well as sections prepared from paraffin-embedded cell line were stained at the Johns Hopkins Oncology Tissue Immunostaining Core Facility, a Clinical Laboratory Improvement Amendments (CLIA)-certified lab, using an automated IHC system (Leica BOND-MAX).

### Animal Use and Care

The xenografts were generated as published previously (27). The animals were maintained in accordance with guidelines of the American Association of Laboratory Animal Care and a research protocol approved by the Johns Hopkins University Animal Use and Care Committee.

### Colony Formation Assays

HNSCC cell lines were seeded in a 6-well plates at a density of 3x10^3^. After 24h, the cells were treated with LRRK2-IN-1 or DMSO (served as control). The growth of cell colonies was monitored for up to 14 days. Colonies were fixed and stained with 4% methylene blue (Sigma, Cat# M9140) in 50% methanol. Counting of colonies formed was carried out in ten fields and representative images were photographed at 2.5x magnification. All experiments were performed in triplicate.

### Soft Agar Assay

Cells were seeded (1x10^5^ cells/well) in a 0.35% agar (Difco, Cat# 214220) mixed with culture medium (on the top of 0.5% noble agar with medium) in 6-well plates. The medium was changed every 3 days and cells were cultured at 37°C for 14 days. The images were captured and analyzed using Image J software. Each experiment was set in triplicate.

### Gene Set Enrichment Analysis

Gene expression data was retrieved from TCGA-HNSC dataset. Hallmark pathways gene sets were obtained from The Molecular Signatures Database (MSigDB). NOTCH signaling gene signatures were obtained from 3 additional pathway databases: Kyoto Encyclopedia of Genes and Genomes (KEGG), Reactome and Pathway Interaction Database (PID). Enrichment scores were calculated (ES) using ssGSEA function GSVA R package with default parameters (28). The ES were MAD scaled, median centered and clipped to the range (–4, 4). Pearson correlation was used to select gene sets correlative with DCLK1mmRNA expression (r>0.4). Heatmaps and bar plots were created using matplotlib python package (v2.0.0).

### iPANDA Pathway Analysis

iPANDA software suite for analysis of intracellular signaling pathway activation based on transcriptomic data was used to estimate the level of the NOTCH pathway activation. Gene expression data was retrieved from TCGA and GEO databases. Data preprocessing and normalization steps were performed in R version 3.1.0 using DEseq package from Bioconductor. Normalized gene expression data were loaded into iPANDA algorithm (29, 30), and Pathway Activation Score (PAS), a value which serves as a quantitative measure of differential pathway activation, was estimated for NOTCH pathway. NOTCH signaling axis was obtained from the four different pathway databases: MSigDB, KEGG, Reactome, and PID.

### Statistical Analysis

Differences between two groups were analyzed by Student’s *t* test using Graphpad Prism software (version 9) (La Jolla, CA). All P values were based on two-sided tests. The significance level was defined as 0.05. Survival analysis was performed using Kaplan-Meier model and significance was determined using a two-sided log-rank test. The level of statistical significance was set at P < 0.05.

## Results

### Pharmacologic Inhibition of DCLK1 Leads to Reduced Proliferation, Migration, Infiltration, Colony Formation and Anchorage Independent Growth

**Figure 1:**
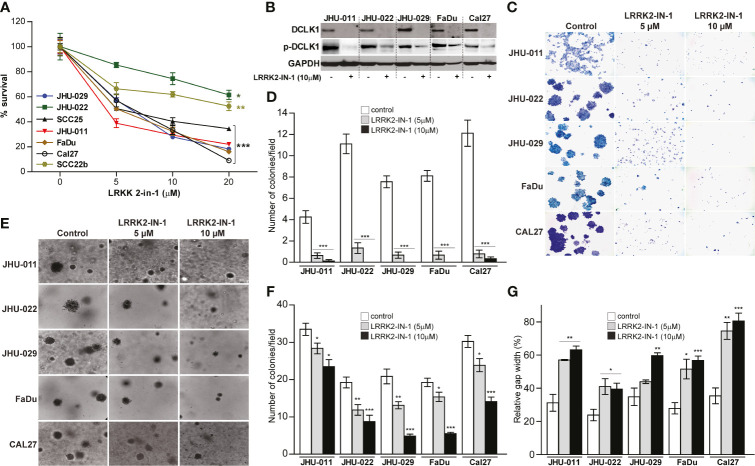
Pharmacologic inhibition of DCLK1 leads to reduced proliferation, migration, infiltration, colony formation and anchorage independent growth. **(A)** Seven HNSCC cell lines were treated with either indicated concentrations of LRRK2-IN-1 or DMSO (solvent) for 72 hours. Relative cell viability was determined using an Alamar Blue assay (*p < 0.05, **p < 0.01 and ***p < 0.001). **(B)** Cell lysates were collected at end point from cells treated with 10μM DCLK1 and control cells and analyzed by western blot for the expression of indicated proteins. GAPDH was used a loading control. **(C, D)** Colony formation assay: cells were treated with LRRK2-IN-1 or vehicle for 14 days. Representative images **(C)** and quantification **(D)** of LRRK2-IN-1 mediated reduction of colony formation in HNSCC cell lines are shown. **(E, F)** Soft agar anchorage-independent growth: cells were treated with LRRK2-IN-1 or vehicle for 14 days. Representative images **(E)** and quantification **(F)** of LRRK2-IN-1 mediated decrease of anchorage-independent growth of HNSCC cells are shown. **(G)** Indicated HNSCC cell lines were treated with 10μM LRRK2-IN-1 or DMSO for 72 hours. Cells were trypsinized, counted, plated in triplicate in 6-well plates (1 x 10^6^ cells per well) containing inserts and allowed to attach for 12 h. The inserts were removed and the gap was photographed immediately and after 12 h. Gap area in individual wells was measured using ImageJ and relative gap width percentage was calculated as the gap area at 12 h relative to the gap area at 0 h (*p < 0.05, **p < 0.01 and ***p < 0.001).

In order to assess the impact that DCLK1 has on HNSCC tumorigenesis, seven head and neck cell lines including, JHU-029, JHU-022, SCC25, JHU011, FaDu, Cal27 and SCC22b, were treated with a small molecule kinase inhibitor LRRK2-IN-1, which was reported to potently inhibit DCLK1 kinase activity (31, 32). Treatment with LRRK2-IN-1 (5-20uM) resulted in significantly reduced survival in all treated cell lines in a dose dependent manner (**Figure 1A**). The 50% inhibitory concentrations (IC50) for all cell lines are summarized in **Supplementary Table 1**. As previously reported (31, 33), treatment with LRRK2-IN-1 resulted in a substantial reduction in both total DCLK1 and phospho-DCLK1 expression levels in all HNSCC cell lines analyzed (**Figure 1B**). As previous studies have shown that targeting DCLK1 with kinase inhibitors resulted in significant reduction of clonogenic potential and invasive capacity in pancreatic cancer and renal cell carcinoma cell lines (31, 33, 34), we next assessed the impact of LRRK2-IN-1 induced DCLK1 inhibition on HNSCC cell lines. To assess the effect of LRRK2-IN-1 on long-term cell-growth, colony formation assays were performed with either LRRK2-IN-1 or vehicle treated cell lines. Exposure to LRRK2-IN-1 showed potent suppression of a colony forming ability, markedly reducing the number and size of the colonies in all HNSCC cells analyzed (p-value <0.001) (**Figures 1C, D**). An *in vitro* indicator for tumorigenic potential is the ability of cells to grow in an anchorage-independent environment (35, 36). We thus next carried out soft agar assays to assess the effect of DCLK1 inhibition on the anchorage-independent growth of HNSCC cells. Treatment with LRRK2-IN-1 was sufficient to impair the anchorage-independent growth capability of all tested HNSCC cell lines, leading to a drastic reduction in the number and size of colonies formed in soft agar medium in dose dependent manner (p- value <0.05) (**Figures 1E, F**). To further evaluate the effect of LRRK2-IN-1 on the oncogenic properties of HNSCC cells, wound healing assays were carried out to assess their migration potential. Treatment with LRRK2-IN-1 significantly inhibited the migration of all HNSCC cell lines compared to the untreated cells grown in parallel (**Figure 1G**).

### siRNA Mediated Down Regulation of DCLK1 Leads to Reduced Growth, Invasion and Migration

**Figure 2:**
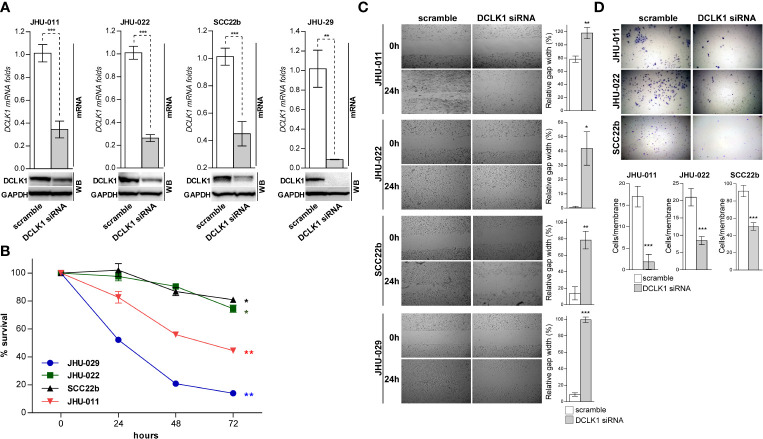
siRNA mediated down regulation of DCLK1 leads to reduced growth, invasion and migration. **(A)** DCLK1 expression was knocked down in four indicated HNSCC cells using siRNA. Cell transfected with scramble non-targeting siRNA were used as controls. mRNA and whole cell lysates were prepared from DCLK1-depleted and control cells and analyzed by RT-PCR (top) and western blot (bottom) for the DCLK1 expression on RNA and protein levels. **(B)** DCLK1 depleted and control cells were plated at equal numbers in triplicates and relative cell viability was determined using an Alamar Blue assay at indicated times points. **(C)** DCLK1 depleted and control cells were trypsinized, counted, plated in triplicate in 6-well plates (1 x 10^6^ cells per well) containing inserts and allowed to attach for 12 h. The inserts were removed and the gap was photographed immediately and after 12 h. Right panels - gap area in individual wells was measured using ImageJ and relative gap width percentage was calculated as the gap area at 12 h relative to the gap area at 0 h (*p < 0.05, **p < 0.01 and ***p < 0.001). **(D)** DCLK1 depleted and control cells were trypsinized, counted and cultured (1×10^4^ cells per well) in triplicate into the transwell chambers. After 24 h the membranes were stained with crystal violet and cells that had migrated through the membrane were counted. Top panel - representative images are shown. Bottom panel - the average number of cells per field that migrated through the membrane is shown as a bar chart (***p < 0.001).

While LRRK2-IN-1 potently suppresses DCLK1 kinase activity and inhibits its mRNA and protein expression (31–33), it was suggested that LRRK2-IN-1 may have potential off-target affinity for other kinases (37, 38), which complicates assigning specific activities to DCLK1. As such, in order to confirm that the phenotype observed in HNSCC cell lines treated with LRRK2-IN-1 indeed resulted from a direct inhibition of DCLK1 activity, we utilized a DCLK1 specific small interfering RNA (siRNA) to reduce its expression in selected HNSCC cell lines (JHU-011, JHU-022, JHU-029 and SCC22b) with high endogenous DCLK1 expression. Introduction of siRNA resulted in substantial DCLK1 expression knock-down in all four HNSCC cells, compared to a scrambled siRNA transfection (**Figure 2A**). Similarly to LRRK2-IN-1, DCLK1 knock-down induced a significant inhibition of cell viability (p-value <0.001) (**Figure 2B**), and attenuated migration compared to the control cells treated (**Figure 2C**). Furthermore, Boyden chamber assays were carried out in 3 HNSCC cell lines to determine the effect of DCLK1 depletion on their invasion potential. DCLK1 knock-down substantially decreased the invasion of all 3 cell lines analyzed (**Figure 2D**), further supporting the role of DCLK1 in regulating the tumorigenic potential of HNSCC cell lines *in vitro*.

### DCLK1 Overexpression Induces Enhanced Cell Proliferation and Upregulation of Key Stem Cell Biomarkers

**Figure 3:**
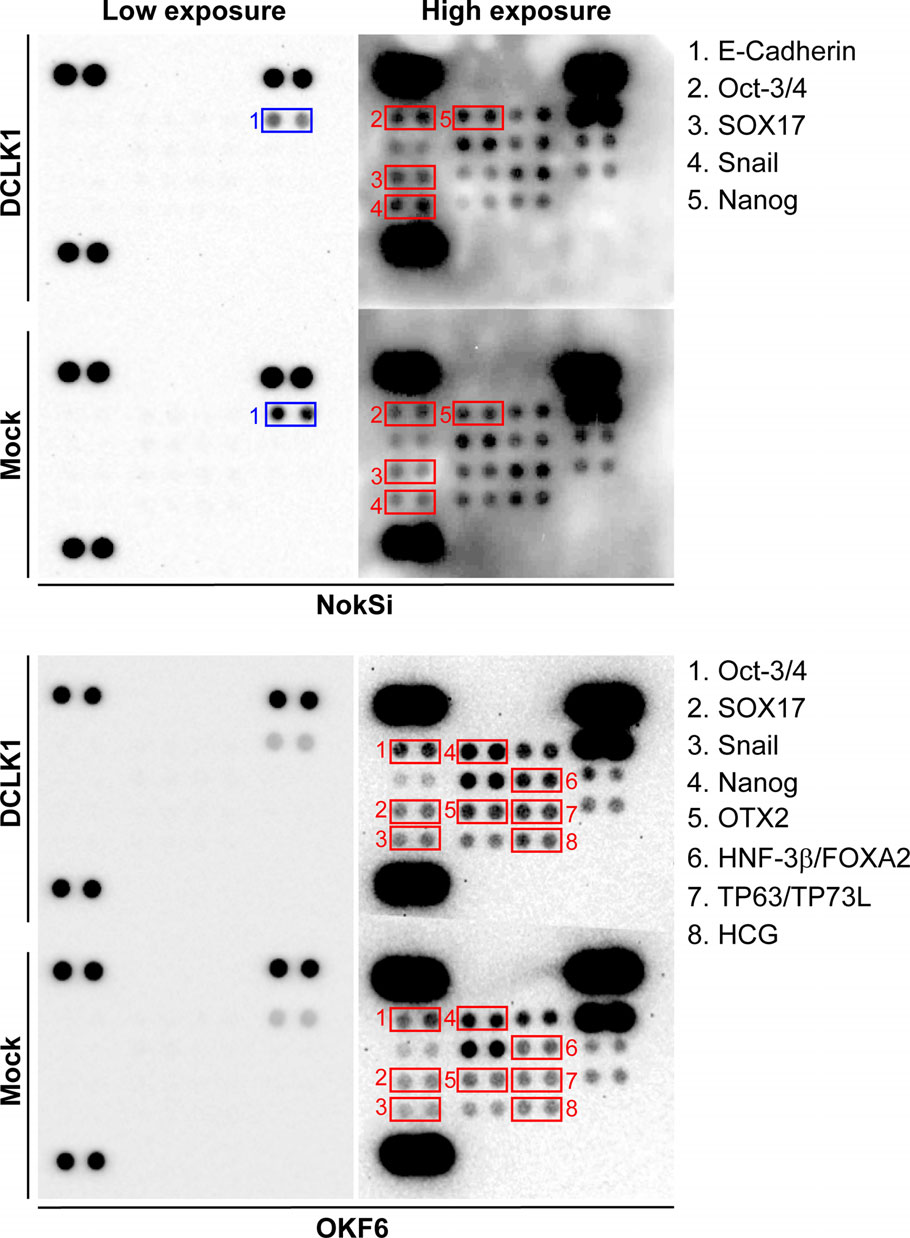
DCLK1 overexpression induces upregulation of key stem cell biomarkers. Human stem cell antibody arrays were performed using whole lysates collected from DCLK1 overexpressing and control NokSi and OKF6 cell lines. Upregulated stem cells markers in the DCLK1 cell lines were highlighted in red boxes. Downregulated E-cadherin in NokSi cells expressing DCLK1 was highlighted in blue boxes.

The opposite effect on cell viability was observed after DCLK1 overexpression in normal oral keratinocytes cell lines with low basal DCLK1 expression, OKF6 and NOKSI, where higher proliferation was observed in DCLK1 expressing cells (**Supplementary Figure 1**). As DCLK1 has been universally identified as a cancer stem cell (CSC) marker essential for maintaining cancer stemness and promotion of cancer initiation and metastasis in many types of cancers (10–12, 39, 40), we next sought to find out whether DCLK1-induced cell proliferation was associated with the increased expression of known stemness biomarkers. We applied a human pluripotent stem cell antibody array using lysates extracted from OKF6 and NOKSI cell lines transfected with either DCLK1 or empty vector (**Figure 3**). Notably, DCLK1 overexpression resulted in elevated expression of OCT-3/4, SOX17, Snail, and Nanog in both OKF6 and NOKSi cells. Furthermore, the expression levels of additional stemness markers, such as OTX2, HNF-3β/FOXA3, TP63/TP73L and HCG, were upregulated in OKF6 cells, whereas NokSi cell line expressing DCLK1 also exhibited reduced level of E-cadherin protein, associated with loss of cell-cell adhesion, proliferation and survival (41). These observations suggest that DCLK1 expression may endow normal cells with greater self-renewal ability and initiate their tumor stem cell function.

### DCLK1 Correlates With NOTCH Pathways in HNSCC Tumors

**Figure 4:**
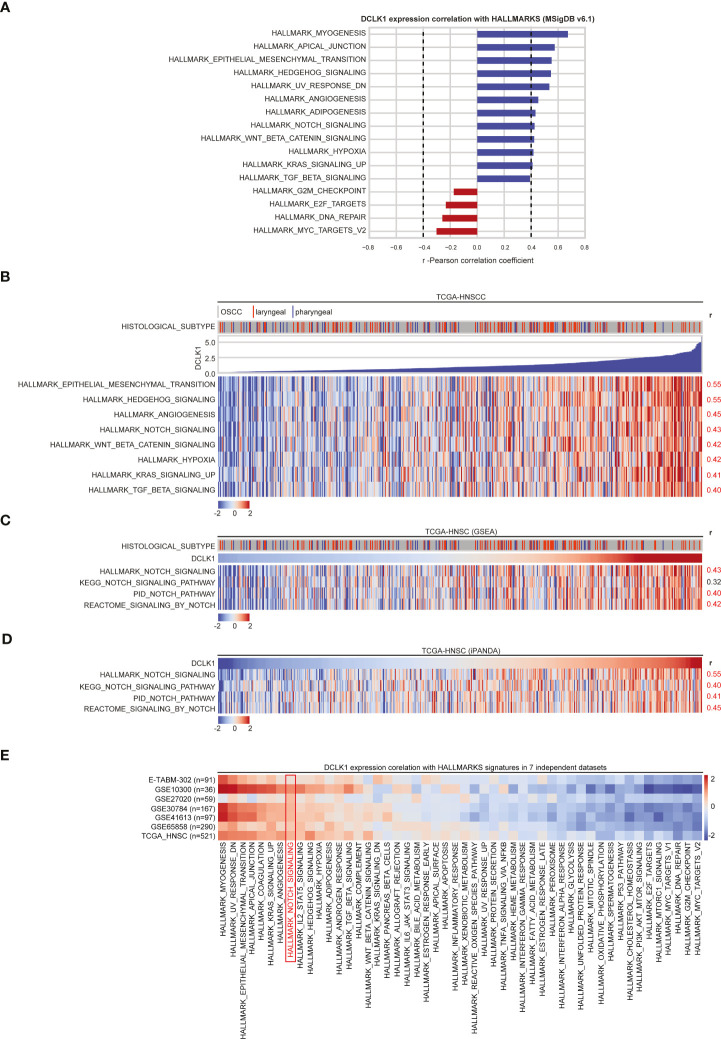
DCLK1 correlates with NOTCH pathways in HNSCC tumors. **(A)** GSEA results of MSigDB Hallmark of cancer gene sets that correlate with DCLK1 mRNA expression in TCGA-HNSC transcriptomic dataset. Blue bars indicate a positive correlation with DCLK1 mRNA expression, whilst red bars indicate negative correlations. Dashed lines indicate Pearson correlation coefficient thresholds for inclusion of the pathways for further analysis. **(B)** Heatmap demonstrates Hallmark pathways that positively correlate (r > 0.4) with DCLK1 mRNA expression in TCGA-HNSC transcriptomic dataset. **(C)** GSEA was performed using NOTCH signaling axis genes derived from 4 pathway databases: KEGG, Reactome, PID, and Hallmark. A positive correlation between DCLK1 mRNA expression and estimated NOTCH signaling enrichment using each database is indicated by r-values on the right. **(D)** iPANDA software suite for analysis of intracellular signaling pathway activation based on transcriptomic data was used to estimate the level of the NOTCH signaling pathway in TCGA-HNSC dataset. NOTCH axis gene sets were derived from 4 pathway databases listed in **(C)**. TCGA transcriptomic data from non-tumorous samples was used as a reference after proper normalization. **(E)** GSEA querying of the hallmark gene sets using the transcriptomic profiles from TCGA-HNSC and 7 additional independent cohorts of HNSCC patients obtained from either NCBI GEO or ArrayExpress databases was performed. For each pathway, a positive correlations with DCLK1 mRNA expression are indicated in red, whilst negative correlations are shaded in blue.

To further ascertain the role of DCLK1 in HNSCC tumorigenesis, we analyzed gene expression profiles obtained from the TCGA-HNSC dataset. Gene set enrichment analysis (GSEA) querying of the hallmark gene sets within the MSigDB (42) revealed that higher DCLK1 mRNA expression positively correlates (r-Pearson correlation coefficient > 0.4) with a number of hallmark pathways associated with cancer proliferation, survival and stemness maintenance, such as the epithelial mesenchymal transition (EMT), Hedgehog, angiogenesis, WNT/β-catenin, KRAS, TGFβ, and NOTCH signaling networks (**Figures 4A, B**). Notably, these correlations were independent of tumor histological subsites (**Figure 4B**), and are in line with previous reports suggesting that DCLK1 is involved in regulating of the NOTCH, KRAS, WNT, and TGFβ pathways to promote progression, EMT, and self-renewal in colorectal and pancreatic cancer cells (11, 13, 43, 44). As the NOTCH signaling axis has a recognized role in the pathogenesis of HNSCC tumors, we focused on investigating the role of DCLK1 in NOTCH pathway regulation.

To this end, we first performed gene set enrichment analysis using the NOTCH signaling axis derived from 3 additional pathway databases: Kyoto Encyclopedia of Genes and Genomes (KEGG), Reactome, and Pathway Interaction Database (PID) curated by NCI/Nature. A positive correlation between DCLK1 expression and NOTCH signaling was supported by all pathway databases used for the analysis (**Figure 4C**). Since pathway-based algorithms such as GSEA and its extensions rely solely on gene enrichment statistics treating pathways as unstructured sets of genes, the In silico Pathway Activation Network Decomposition Analysis (iPANDA) algorithm (29, 30) was pursued to predict differential activation of the NOTCH pathway in the HNSCC tumor samples using non-neoplastic mucosa specimens as a reference. Notably, the analysis was highly reflective of the GSEA results, albeit with slightly elevated correlation scores between DCLK1 expression and NOTCH signaling activation (**Figure 4D**).

Moreover, GSEA querying of the hallmark gene sets using the array-based transcriptomic profiles from 7 additional independent cohorts of HNSCC patients obtained from either NCBI GEO (GSE10300, GSE27020, GSE30784, GSE41613, GSE65858) or ArrayExpress databases (E-TABM-302), confirmed a positive correlation between DCLK1 expression and the NOTCH pathway in all 7 publicly available data sets analyzed (**Figure 4E**).

### DCLK1 Influences NOTCH Signaling *In Vitro*

**Figure 5:**
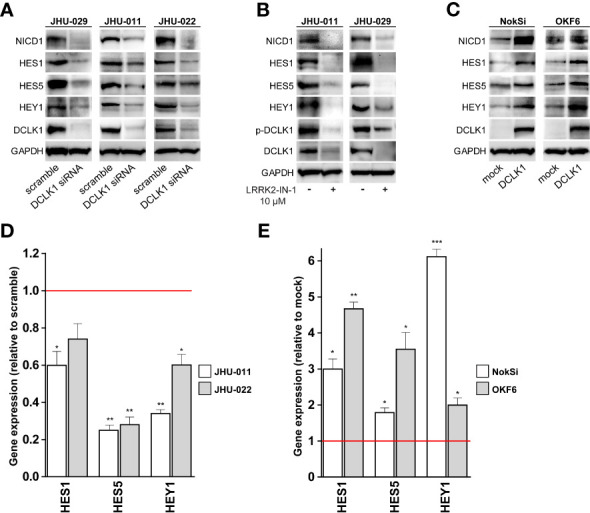
DCLK1 influences NOTCH signaling in *vitro*. **(A)** DCLK1 expression was knocked down in three indicated HNSCC cell lines using siRNA. Cell transfected with scramble non-targeting siRNA were used as controls. Whole cell lysates were prepared from DCLK1-depleted and control cells and analyzed western blot for the expression of indicated proteins. **(B)** Two HNSCC cell lines were treated with either LRRK2-IN-1 or DMSO (solvent) for 72 hours. Cell lysates were collected at end point and analyzed by western blot for the expression of indicated proteins. GAPDH was used a loading control. **(C)** Forced expression of DCLK1 in two normal human oral keratinocytes cell lines expressing low level of endogenous DCLK1. Cell lysates were collected from DCLK1-expressing cells and control cells infected with empty vector and analyzed by western blot for the expression of indicated proteins. GAPDH was used a loading control. **(D)** RT-PCR results of NOTCH target genes in DCLK1-depleted HNSCC cells, demonstrating significant reduction relative to the control cells (red line). **(E)** RT-PCR results of NOTCH target genes in DCLK1 overexpressing normal human oral keratinocytes cell lines, demonstrating significant elevation relative to the control cells (red line). (*p < 0.05, **p < 0.01 and ***p < 0.001).

The NOTCH signaling pathway is known to maintain stem cells through transcriptional activation of HES/HEY family members to repress tissue-specific transcription factors (45). Interestingly, siRNA mediated DCLK1 depletion in 3 HNSCC cell lines (JHU-011 JHU-022 and JHU-029) substantially downregulated the expression levels of the NOTCH1 intracellular domain (NICD) and its downstream targets, such as HES1, HES5 and HEY1 (**Figure 5A**), confirming that NOTCH signaling was functionally inhibited. Notably, the same pattern was observed when DCLK1 was inhibited by LRRK2-in-1 (**Figure 5B**). Conversely, overexpression of DCLK1 in normal human oral keratinocytes cell lines (OKF6 and NOKSi), resulted in elevated expression of both cleaved NOTCH1 as well as of HES/HEY proteins (**Figure 5C**). RT-PCR analysis confirmed that expression of HES1, HES5 and HEY1 on the RNA level was lower in cells treated with DCLK1 siRNA, compared to the cells treated with the scrambled control (**Figure 5D**). Moreover, their expression levels were higher in cells where DCLK1 was overexpressed, compared to the cells transfected with the control vector (**Figure 5E**). Taken together, these observations further support the role of DCLK1 in the NOTCH signaling pathway regulation.

### Inhibition of NOTCH Signaling Decreases DCLK1 Expression *In Vitro* and *In Vivo*

To test the effect of NOTCH pathway blockade on DCLK1, JHU-011 cell line (which expresses high level of NICD1, reflecting active NOTCH signaling) was treated with BMS-906024, a γ-secretase inhibitor that potently inhibits all 4 NOTCH homologues at nanomolar concentrations (46). Inhibition of NOTCH signaling significantly reduced cell proliferation and was associated with a significant reduction in both NICD1 and DCLK1 expression, as confirmed by IHC analyses (**Supplementary Figure 2A**). Notably, treatment of HNSCC patient-derived xenograft (PDX) model which displays high level of nuclear NICD1 and elevated DCLK1 expression with NOTCH inhibitor, resulted in a similar effect (**Supplementary Figure 2B**). While these observations may suggest a potential feedback mechanism, the role of NOTCH signaling in HNSCC is complex (21), and studies using models bearing tumors from different histological sites and showing various levels of DCLK1 expression are necessary to further delineate a mechanism of DCLK1-NOTCH cooperation.

### Expression of DCLK1 Is Associated With Poor Prognosis in Patients With HNSCC

**Figure 6:**
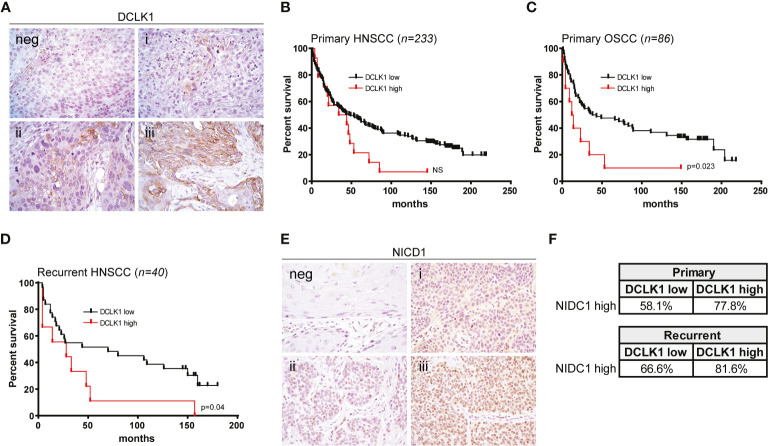
Expression of DCLK1 is associated with poor prognosis in patients with HNSCC. **(A)** Representative staining of DCLK1 in HNSCC tumors: (neg) negative, (i) weak, (ii) moderate, (iii) high expression. **(B)** Kaplan Meier estimates of overall survival based on DCLK1 protein expression status in a cohort of 233 primary HNSCC tumors. Low expression (negative and weak staining) - black line, high expression (moderate and high staining) - red line. **(C)** Kaplan–Meier estimates of overall survival based on DCLK1 protein expression status in a cohort of 86 primary OSCC tumors. Low expression (negative and weak staining) - black line, high expression (moderate and high staining) - red line (p = 0.023). **(D)** Kaplan–Meier estimates of overall survival based on DCLK1 protein expression status in a cohort of 40 recurrent HNSCC tumors (low expression - black line, high expression - red line) (p = 0.04). **(E)** Representative staining of NICD1 in HNSCC tumors: (neg) negative, (i) weak, (ii) moderate, (iii) high expression. **(F)** Table demonstrates a higher percentage of tumors with high NICD1 staining, among patients that also demonstrate higher DCLK1 staining.

To assess the prevalence and clinical implications of DCLK1 expression in HNSCC, were analyzed DCLK1 protein levels by IHC in a tumor microarray (TMA) comprised of 273 cancer biopsies (233 patients presented with a primary tumors and 40 patients presented with recurrent malignancies) (**Figure 6A**). Among patients with primary malignancy, the average age was 59.3 years with 74.2% of the population being male. Clinicopathological characteristics of the patients included in the TMA are summarized in **Table 1**. The median follow-up was 74 months (range 0–219). DCLK1 expression was detected in 23% of patients with primary HNSCC, and higher expression levels showed a trend with poor overall survival, as shown by the Kaplan–Meier survival curves using cox regression analysis (p = 0.07) (**Figure 6B**). Since our cohort was comprised of patients with different histologic subtypes of HNSCC, we evaluated the association between DCLK1 expression and clinical outcome in patients with oral (n = 86), oropharyngeal (n=65), laryngeal (n=56), and hypopharyngeal (n=15) carcinoma. The DCLK1 demonstrates cytoplasmic expression, and the localization, distribution, and intensity of staining was consistent across the anatomical sites (**Supplementary Figure 2**). Notably, while DCLK1 expression was significantly associated with worse overall survival (p = 0.023) in oral squamous cell carcinoma (OSCC), which represents the most common subtype of HNSCC (47) (**Figure 6C**), no differences were observed in laryngeal and/or oropharyngeal SCC. The survival differences for hypopharyngeal SCC remained statistically insignificant since the sample size was too small. We next evaluated DCLK1 status in a cohort of 40 patients with recurrent HNSCC (**Table 2**). Similar to the newly diagnosed patients, the average age was 60.3 years with 77.5% of the cohort being male. Unlike the newly diagnosed patients, a larger subset (32.5%) of recurrent HNSCCs was DCLK1 positive. Univariate analysis of DCLK1 expression in these patients showed that higher DCLK1 expression level was significantly (p=0.04) associated with worse survival (**Figure 6D**). We next analyzed the same TMA for NIDC1 expression (**Figure 6E**). Although statistical significance was not reached, in patents with both primary and recurrent disease, there was a trend towards higher NIDC1 expression in tumors with elevated DCLK1 levels (**Figure 6F**).

**Table 1:** Demographic and clinical characteristics of patients with primary HNSCC.

	All patients	DCLK1 negative	DCLK1 positive
**Marker**			
DCLK1	233	181	52
**Age at diagnosis**			
Mean	59	59	60
Range	25-96	25-96	31-88
**Gender**			
Female	60	51	9
Male	173	130	43
**Primary tumor site**			
Hypopharynx	15	11	4
Larynx	56	36	11
Oral	86	75	20
Oropharynx	65	51	14
Others	11	8	3
**T stage**			
T1	59	48	11
T2	62	48	14
T3	46	33	13
T4	52	39	13
T x	4	4	0
TNA	10	9	1
**N stage**			
N0	93	70	23
N1	29	24	5
N2	10	8	2
N2a	8	4	4
N2b	48	39	9
N2c	30	23	7
N3	3	2	1
Nx	1	1	0
N NA	11	10	1

**Table 2:** Demographic and clinical characteristics of patients with recurrent HNSCC.

	All patients	DCLK1 negative	DCKL1 positive
**Marker**			
DCLK1	40	27	13
**Age at recurrence**			
Mean	60.3	59.5	61.0
Range	34-88	34.88	34-86
**Gender**			
Female	9	5	4
Male	31	23	8

## Discussion

HNSCC is a heterogeneous disease that includes squamous cell carcinomas (SCC) of the oral cavity, pharynx, and larynx. The disease is more prevalent in males, and those individuals who smoke or chew tobacco and/or consume alcohol are at much higher risk for HNSCC (48). The growth of HNSCC is maintained by a population of cancer stem cells (CSCs) which possess unlimited self-renewal potential and induce tumor regrowth if not completely eliminated by therapy (49). DCLK1 is a validated novel CSC marker in the gastrointestinal tract and there is emerging evidence for an equivalent role in other cancers (10–15), including HNSCC (16–18). However, while DCLK1 has been actively studied in several solid malignancies, there is limited data on its role in HNSCC tumerogenesis (49, 50). In this study, we used bioinformatics, immunohistochemistry, and cell physiology assays in combination with overexpression and siRNA-knockdown to study the role of DCLK1 in head and neck cancer.

Our results demonstrate that silencing of DCLK1, through either pharmacologic inhibitor or siRNA, significantly inhibited key neoplastic characteristics of the head and neck cancer cell lines, such as proliferation, migration, and invasion, whereas overexpression of DCLK1 in normal keratinocytes induced cell proliferation and survival, and was associated with elevated expression of stemness markers. Although this aligns with prior research in other solid tumors; for instance, increased DCLK1 has been reported to induce pancreatic, colorectal and ovarian cancer cell migration and invasion (51–53), the mechanisms through which DCLK1 exerts these effects in HNSCC remain largely unknown (17).

It is important to note that human DCLK1 consists of two primary isoforms with a shared kinase domain, generated by alternative promoter usage and alternative splicing: long (DCLK1-L, NM_004734) and short (DCLK1-S, NM_001195415) variant, with molecular weight of ~80–82 and ~45–50 KDa, respectively (54). In the recent decade, multiple studies have confirmed clinical value of DCLK1-L expression in tumor progression. Although more recently it has been suggested that DCLK1-S isoform could play different function in driving aggressive behavior of tumor cells (33, 55), the molecular evidence regarding isoform-specific function is limited and remains highly controversial. Furthermore, these isoforms have been given different names in literature, adding to confusion in the field (56). The PCR assay used in our study targets homologous coding sequence of both long and short isoforms (56), whereas anti-DCLK1 antibody (22–26) used for WB and IHC analyses specifically detects only the 82 KDa isoform according to the manufacturer (see Methods). Although a band that could be interpreted as 52 KDa isoform was seen in most of the HNSCC cell lines (not shown), we relied exclusively on the 82 KDa isoform when analyzing the data. Further studies utilizing isoform-specific primers and antibodies are required to accurately assess a variable expression of various DCLK1 isoforms in HNSCC.

It was suggested that DCLK1 regulates migration, invasion and cell motility *via* activation of EMT (43), an important process for cancer initiation, cancer metastasis, and secondary tumor formation. However, expression of DCLK1 was also associated with impaired DNA repair (57, 58), upregulation of WNT/β-catenin (59, 60), RAS (13, 61), PI3K/AKT (62) and VEGF (63, 64) signaling, suggesting a multifaceted role of DCLK1 in cancer initiation and progression. Concordantly, our analysis of TCGA-HNSC dataset indicates that in HNSCC, tumors expressing higher DCLK1 mRNA levels were enriched for pathways that have been implicated in the EMT and CSC maintenance (such as NOTCH, WNT, TGFβ, and hedgehog signaling), as well as for RAS and angiogenesis cascades.

The NOTCH signaling network is particularly noteworthy. Although accumulating data suggest that NOTCH is one of the most frequently altered pathways in HNSCC, only a few studies have directly examined the regulation of the NOTCH pathway in the context of head and neck cancer (21). Notably, our thorough bioinformatics analysis of hundreds of HNSCC tumors obtained from several publicly available datasets confirmed a positive correlation between DCLK1 expression and NOTCH signaling activation. Furthermore, inhibition of DCLK1 in HNSCC cells resulted in a substantial decrease in NOTCH1 activation and expression of canonical NOTCH target genes (HES1, HES5 and HEY1), whereas overexpression of DCLK1 in normal keratinocytes induced an opposite effect. This data is well in line with previous findings in colon cancer, showing that depletion of DCLK1 may inhibit NOTCH1 expression *via* upregulation of microRNA-144 (44). While concurrent decrease or increase of NOTCH pathway elements and DCLK1 was also reported in crypt epithelial stem cells following radiation injury (65), epithelial tuft cells (66), and enteric infection (67), further supporting the role of DCLK1 in NOTCH signaling regulation, additional studies are warranted to decipher the mechanism underlying a cross-talk between DCLK1 and NOTCH activities in HNSCC.

Our analyses of a TMA containing HNSCC tumors obtained from 233 newly diagnosed and 40 recurrent patients revealed that high DCLK1 staining was strongly associated with decreased survival, confirming a previous report showing that DCLK1 mRNA level correlated with poor clinical outcome in a smaller cohort of NSCC patients that underwent surgery and postoperative radiotherapy (17). While DCLK1 expression was independent of HPV status, histological site and stage, in our cohort of patients with primary tumors, DCLK1 was significantly associated with survival only in OCSS patients, contradicting a previous report from another group showing that statistical significance was achieved only in oropharyngeal SCC (17). This discrepancy may be explained by a different composition of patients included in each study group, and higher ethnical heterogeneity among US HNSCC patients. However, further studies in a larger cohort of clinical samples are necessary to address the clinical significance of DCLK1 upregulation at different histological sites.

Notably, the frequency of tumors expressing a high level of NICD1 was increased among patients with elevated DCLK1 expression in both cohorts, further suggesting that in some cases, concurrent expression of DCLK1 and activation of NOTCH signaling may facilitate a rapidly progressing and aggressive tumor phenotype. In most tumor types, NOTCH signaling has been reported be oncogenic. However, early characterization of the genomics landscape found that inactivating mutations of NOTCH1 frequently occur in HNSCC, suggesting that NOTCH1 may also function as a tumor suppressor (21). More recent evidence indicates that NOTCH signaling may be activated in a subset of head and neck tumors, similar to other cancer types, indicating a more complex function in HNSCC (20, 68). It is plausible that a subset of patients in which NOTCH acts as an oncogene may also display overexpressed DCLK1, allowing the tumor to co-opt and amplify mechanisms underlying HNSCC initiation and progression. As evidence accumulates that DCLK1 is important in driving tumorigenesis, its kinase domain represents an obvious site to target for drug discovery efforts.

While our data support the notion that DCLK1 plays important role in HNSCC tumorigenesis *via* NOTCH signaling, crosstalk with components of other pathways may also modulate the NOTCH axis activity in patients with head and neck malignancies (21). As cell lines cannot entirely recapitulate the multifaceted effect of NOTCH signaling in clinically diverse HNSCC tumors from different anatomical sites that may or may not contain oncogenic HPV, and from patients that have had varied levels of tobacco smoke exposure, continuous generation and analysis of patient-derived xenografts and alternative cost-effective preclinical models, such as three-dimensional organoids, may allow characterization of the molecular features that define the contextual functional role of DCLK1 in regulating NOTCH signaling.

Several NOTCH-targeted treatment paradigms are currently being tested in preclinical studies and clinical setting. Moreover, the anti-oncogenic effects induced by DCLK1 inhibition in several solid tumors, furnished a logical foundation for the development of therapeutic reagents targeting its kinase activity. The recent development of a potent and highly specific DCLK1 inhibitor (69) suggests that both targets can be optimally exploited for the treatment of patients with HNSCC and other cancers driven by DCLK1 and NOTCH pathway alterations.

## Data Availability Statement

The original contributions presented in the study are included in the article/**Supplementary Material**. Further inquiries can be directed to the corresponding authors.

## Ethics Statement

The studies involving human participants were reviewed and approved by Johns Hopkins University Institutional Review Board (IRB). The patients/participants provided their written informed consent to participate in this study. Samples were collected from the Johns Hopkins University Head and Neck Database (HAND) following Institutional Review Boards (IRB) approved protocols. The animal study was reviewed and approved by Johns Hopkins University Animal Use and Care Committee.

## Author Contributions

Conceptualization: EI, EB, DS, and AC. Methodology: EI, EB, DS, AC, TS, AZ, MK, and IO. Validation: EB, MK, IO, NK, EI, and AZ. Formal analysis: EB, EI, MK, IO, LR, and VM. Investigation: EB, JT, TS, and LS. Resources: LR, NA, AP, AR, and PS. Data curation: LR, MK, AP, AR, and PS. Writing - original draft preparation: EI, EB, JT, AC, and NA. Writing - review and editing: EI, DS, EB, JT, NA. AC, PS, VM, and AR. Visualization: MK, IO, NK, EI, EB, and AZ. Supervision: EI, DS, and AC. Funding acquisition: EI, DS, and NA. All authors contributed to the article and approved the submitted version.

## Funding

This work was supported by NIDCR/NIH grant R01DE027809, R01DE028674 and Specialized Programs of Research Excellence in Human Cancers (SPORE) P50DE019032.

## Conflict of Interest

MK, AZ, and IO are affiliated with Insilico Medicine.

The remaining authors declare that the research was conducted in the absence of any commercial or financial relationships that could be construed as a potential conflict of interest.

## Supplementary Material

Supplementary Figure 1 DCLK1 overexpressing NokSi and OKF6 cell lines (gray bars) and control cells (while bars) were plated at equal numbers in triplicates and relative cell viability was determined using an Alamar Blue assay at 72 hours after transfection.

Supplementary Figure 2
**(A)** Left - JHU-011 cells were treated with either DMSO or 10nM BMS-906024 in triplicates for 96 hours and relative cell viability was determined using an Alamar Blue assay. Right - immunohistochemical staining of the FFPE cell pellet blocks generated from JHU-011 cells treated with either 10nM BMS-906024 or DMSO for 96 hours. **(B)** Left - HNSCC PDX model harboring tumor with high level of NICD1 and DCLK1 expression was treated with either BMS-906024 (5 mg/kg; per os) or vehicle. Graph shows the average tumor volume for five animals ± SD. Right - tumor sections derived from xenograft model treated with either BMS-906024 or vehicle stained for NICD1 and DCLK1.

Supplementary Figure 3 Representative staining of high DCLK1 expression in oral squamous cell carcinoma (OSCC), oropharyngeal squamous cell carcinoma (OPSCC) and laryngeal squamous cell carcinoma (LSCC).

Supplementary Table 1 Table summarizes IC50 values for 7 HNSCC cell lines treated with increasing concentrations of LRRK2-IN-1.
