# Supplementary figures and images for: Doublecortin-Like Kinase 1 (DCLK1) Is a Novel NOTCH Pathway Signaling Regulator in Head and Neck Squamous Cell Carcinoma
Source: Front Oncol. 2021 Jul 16;11:677051. doi: 10.3389/fonc.2021.677051 (PMC8323482; doi:10.3389/fonc.2021.677051)

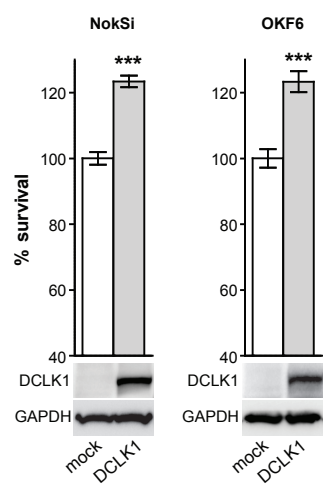

**Supplementary Figure 1**

Supplement: Supplementary Figure 1 — DCLK1 overexpressing NokSi and OKF6 cell lines (gray bars) and control cells (while bars) were plated at equal numbers in triplicates and relative cell viability was determined using an Alamar Blue assay at 72 hours after transfection. [file DataSheet_1.pdf]

**A.**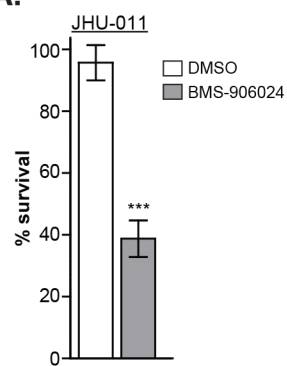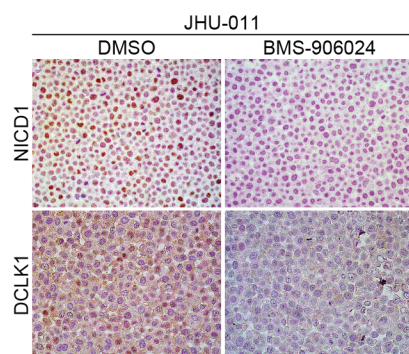**B.**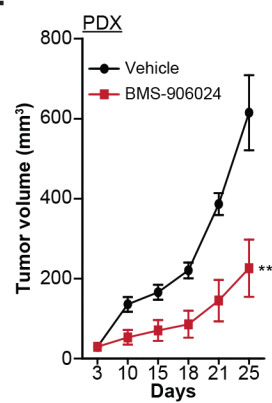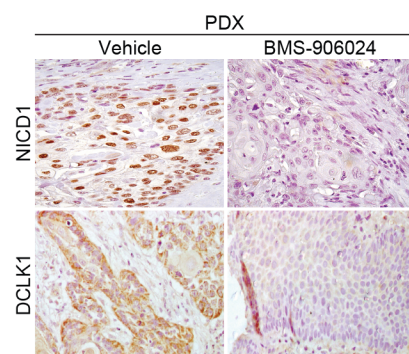**Supplementary Figure 2**

Supplement: Supplementary Figure 2 — (A) Left - JHU-011 cells were treated with either DMSO or 10nM BMS-906024 in triplicates for 96 hours and relative cell viability was determined using an Alamar Blue assay. Right - immunohistochemical staining of the FFPE cell pellet blocks generated from JHU-011 cells treated with either 10nM BMS-906024 or DMSO for 96 hours. (B) Left - HNSCC PDX model harboring tumor with high level of NICD1 and DCLK1 expression was treated with either BMS-906024 (5 mg/kg; per os) or vehicle. Graph shows the average tumor volume for five animals ± SD. Right - tumor sections derived from xenograft model treated with either BMS-906024 or vehicle stained for NICD1 and DCLK1. [file DataSheet_2.pdf]
