# Supplementary material for: Doublecortin-Like Kinase 1 (DCLK1) Is a Novel NOTCH Pathway Signaling Regulator in Head and Neck Squamous Cell Carcinoma
Source: Front Oncol. 2021 Jul 16;11:677051. doi: 10.3389/fonc.2021.677051 (PMC8323482; doi:10.3389/fonc.2021.677051)
Supplement: Supplementary Figure 3 — Representative staining of high DCLK1 expression in oral squamous cell carcinoma (OSCC), oropharyngeal squamous cell carcinoma (OPSCC) and laryngeal squamous cell carcinoma (LSCC). [file DataSheet_3.pdf]

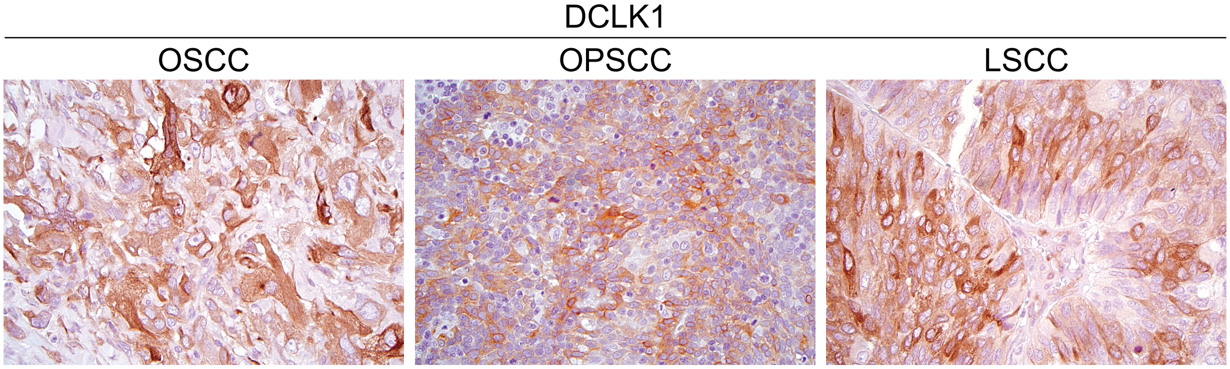

OSCC - oral squamous cell carcinoma
OPSCC - oropharyngeal squamous cell carcinoma
LSCC - laryngeal squamous cell carcinoma

Supplementary Figure 3
