# Supplementary material for: Doublecortin-Like Kinase 1 (DCLK1) Is a Novel NOTCH Pathway Signaling Regulator in Head and Neck Squamous Cell Carcinoma
Source: Front Oncol. 2021 Jul 16;11:677051. doi: 10.3389/fonc.2021.677051 (PMC8323482; doi:10.3389/fonc.2021.677051)
Supplement: Supplementary Table 1 — Table summarizes IC50 values for 7 HNSCC cell lines treated with increasing concentrations of LRRK2-IN-1. [file DataSheet_4.pdf]

HNSCC cell line	IC50 (μM)
**JHU11**	3.0
**SCC25**	4.4
**FaDu**	6.0
**JHU29**	7.3
**Cal27**	7.4
**JHU22**	22.2
**SCC22b**	23.4

Supplementary Table 1
